# Pseudoxanthoma Elasticum of the Skin with Involvement of the Oral Cavity

**DOI:** 10.1155/2013/490785

**Published:** 2013-05-18

**Authors:** Flávia Sayuri Matsuo, Alceu Luiz Camargo Villela Berbert, Sônia Antunes de Oliveira Mantese, Adriano Mota Loyola, Sérgio Vitorino Cardoso, Paulo Rogério de Faria

**Affiliations:** ^1^Department of Morphology, Laboratory of Histology, Institute of Biomedical Science, Federal University of Uberlândia, Avenue Pará 1720, Block 2B, Room 2B-256, 38405-320 Uberlândia, Minas Gerais, Brazil; ^2^Department of Dermatology, School of Medicine, Federal University of Uberlândia, Avenue Pará 1720, Block 2U, Room 23, 38405-302 Uberlândia, Minas Gerais, Brazil; ^3^Department of Oral Pathology, School of Dentistry, Federal University of Uberlândia, Avenue Pará 1720, University Hospital, Room 2K07, 38405-302 Uberlândia, Minas Gerais, Brazil

## Abstract

Pseudoxanthoma elasticum (PXE) is an inherited multisystemic disease of elastic fibers that primarily affects the skin and retina. A case of primary PXE of the skin with late involvement of the upper lip is reported. A 55-year-old woman with a previous diagnosis of PXE affecting her skin developed a lesion on her lower lip. An oral examination identified a yellowish macule of undefined limits. A biopsy from her lip was taken and both light and transmission electron microscopies confirmed the presence of fragmented elastic fibers and calcifications on her mucosa, which was compatible with the diagnosis of oral PXE. Since the manifestation of oral PXE is rare in this region, dental practitioners must be aware that this systemic condition may produce oral lesions, which sometimes may mimic other benign diseases of the oral cavity like Fordyce granules. So, the establishment of an appropriate diagnosis is necessary to provide adequate information and attention to the patient.

## 1. Introduction

Pseudoxanthoma elasticum (PXE) is a multisystemic heritable disease characterized by fragmentation and calcification of elastic fibers [1]. It has been associated with ABCC6-mutation gene, which is responsible for encoding an ATP-dependent transmembrane transporter especially in liver and kidneys [2]. However, the exact mechanism governing its occurrence is heretofore unknown [3, 4]. Classical manifestations include cutaneous yellow papules “pseudoxanthomas [*sic*]”, loss of visual acuity, and atherosclerosis, with considerable morbidity and occasional mortality [1, 5]. The prevalence of PXE is estimated range from 1 : 25,000 to 1 : 100,000. However, the real number of cases may be much higher due to the difficulty of accurately establishing its diagnosis [3, 5]. Early PXE detection is extremely important to prevent systemic complications, especially as those seen in the cardiovascular system [6]. Involvement of the oral mucosa has been described in the literature and is potentially useful to the diagnosis of the disease, but little attention has been given in respect to the clinicopathological features of this manifestation in this region [1, 7–9]. 

To the best of our knowledge only six reported cases of oral PXE have been found in the English literature [7, 8, 10–12]. In this sense, it is worth reporting this case, especially to help dental practitioners in recognition of oral PXE lesions and in establishing an early and correct diagnosis of this life-threatening condition. So, this study describes a case of PXE affecting a woman who developed lesions in the oral mucosa during the progression of the disease and also presents detailed information about clinical, microscopic and ultrastructural aspects of the oral and skin lesions.

## 2. Case Report

**Figure 1:**
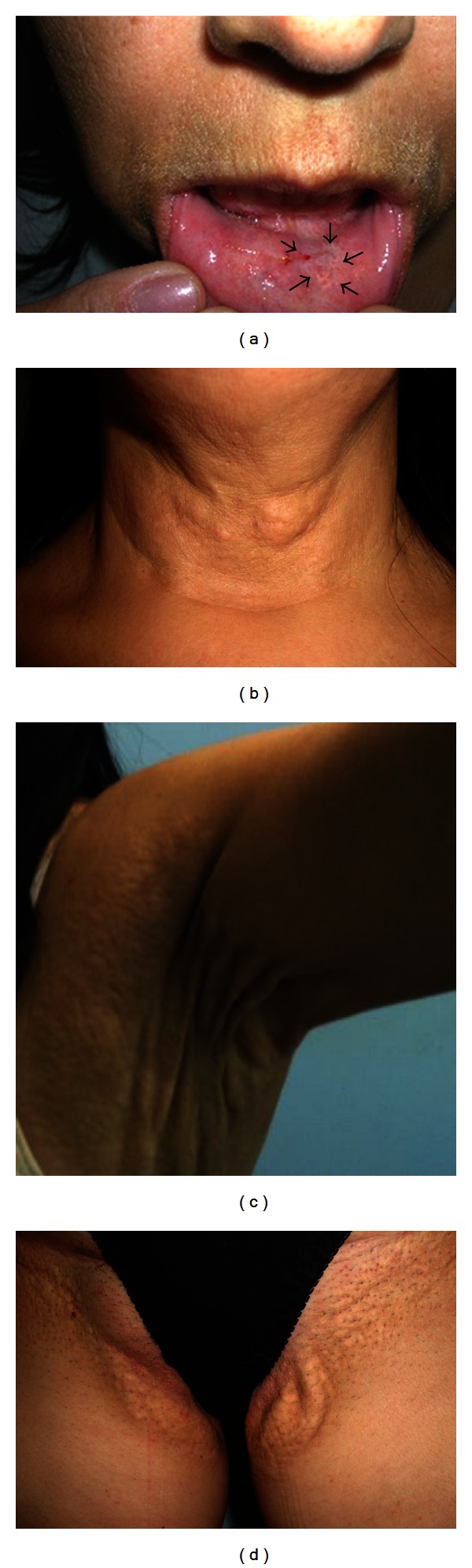
Clinical signs of the lesions. (a) White macule on the lower lip (arrows). (b) Skin lesion in the neck. (c) Skin lesion in the axillary region. (d) Skin lesion in the inguinal region.

A 55-year-old woman presented to our Dermatologic Service informing a previous diagnosis of PXE and looking for treatment for her condition. During the anamnesis, she reported that her condition appeared when she was 16-year old. According to her, at that time, progressive, coalescent yellow papules appeared first on the axillae and after on flexural sites including elbows, neck, and groin (Figures 1(a), 1(b), 1(c), and 1(d)). In respect to the PXE diagnosis, the patient also reported that it was made by a general practitioner. However, no other detailed information was gotten from her during the anamnesis. Moreover, we could not have access to her medical records to confirm all information foregoing. Neither visual loss nor gastrointestinal alterations were noticed by her, except for arterial systemic hypertension, and dyslipidemia, which have been treated by using specific medication. The patient's family history was unremarkable with none of her siblings and first- and second-degree relatives affected by the disease. Clinically, the skin lesions revealed yellowish plaques on the flexural locations including axillary and neck regions. Likewise, oral examination also revealed a yellowish macula on the inner aspect of her lower lip (Figure 1(a)). After dermatological and oral examinations, fragments from the skin and lower lip were taken, routinely processed, and stained with hematoxylin and eosin to confirm the PXE diagnosis. Also, a small piece of the lip lesion was cut out to be analyzed by transmission electron microscopy trying to identify ultrastructural changes in the connective tissue that could suggest PXE. 

**Figure 2:**
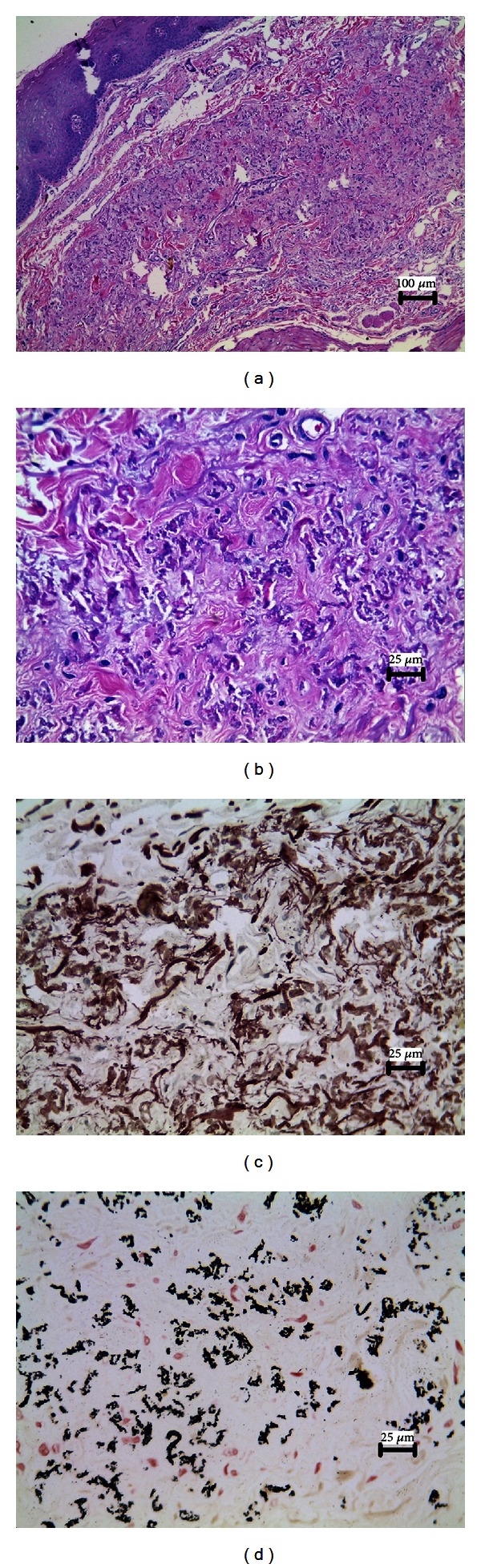
Histopathological aspects of the lesion. (a) Presence of a noncapsulated lesion in the connective tissue (H&E, original magnification ×100). (b) An aggregation of fragmented and basophilic fibers dispersed along with collagen fibers (H&E, original magnification ×400). (c) Orcein staining showing an increased amount of fragmented elastic fibers (original magnification ×400). (d) Von Kossa staining confirming the presence of calcium deposits inside the lesion (original magnification ×400).

As the microscopic features of the skin lesion were similar to the oral one, we will describe herein only those seen in the oral lesion. Microscopically, the lip lesion showed a well-circumscribed, noncapsulated lesion composed of scattered deposits of fragmented and basophilic fibers among collagenous fibers of the connective tissue (Figures 2(a) and 2(b)). Neither inflammation nor signals of any causative agent were observed. Orcein and Von Kossa staining revealed a large amount of shortened, fragmented elastic fibers and deposits of black bodies throughout the lesion, confirming the presence of calcium and phosphorus (Figures 2(c) and 2(d)). 

**Figure 3:**
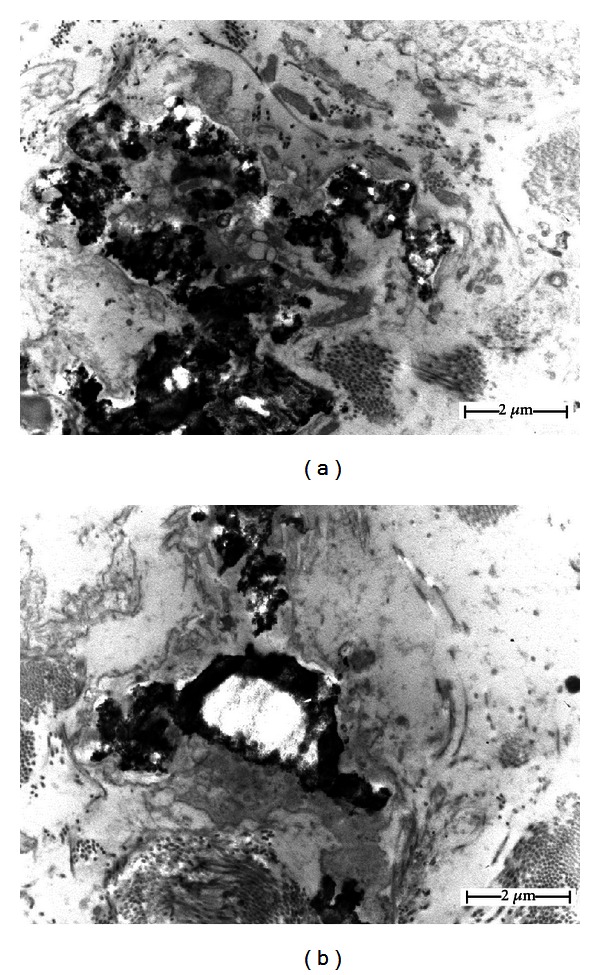
An ultrastructural view of the lesion obtained from the oral cavity. (a) Note the presence of electron-dense calcified bodies in the core of the elastic fibers with some calcifications resulting in ruptures. (b) Observe the presence of calcified material in the center of an elastic fiber.

In addition, by using transmission electron microscopy, numerous aggregates usually large, but sometimes small, electron-dense calcified bodies in the cores of the elastic fibers were identified, resulting in the rupture of some elastic fibers (Figures 3(a) and 3(b)). Based on the clinical features and histopathological and ultrastructural aspects, a diagnosis of oral PXE was carried out. 

Next, the patient was referred to the ophthalmologic department at our institution to be submitted to a funduscopic examination, which in turn revealed the formation of angioid streaks without visual loss. She was also sent to the cardiology and gastroenterology departments to be systemically evaluated, but no abnormalities were found. After that, it was explained to the patient about the nature and expected evolution of her condition, as well as the impossibility of curative treatment. She was also advised to be monitored regularly in an effort to detect early signals of the loss of acuity or secondary choroidal neovascularization (another severe ocular complication), as also to prevent other complications that are likely to develop over the progression of the disease, especially those affecting the gastrointestinal and cardiovascular systems. After three years of followup, both skin and oral lesions have remained stable, as also her ophthalmic condition. Additionally, cardiovascular and gastrointestinal monitoring has not revealed any alterations so far.

## 3. Discussion

PXE, first reported in the literature as a sporadic disease, is nowadays considered to be an inherited genodermatosis disorder of fiber elastics that present an estimated prevalence ranging from 1 : 25,000 to 1 : 100,000 [13]. Women are affected more often than men at ratio of 2 : 1 [6, 8, 14–16]. This disease can affect different populations worldwide, but present a much higher prevalence in Afrikaners living in South Africa [3]. Although PXE can be an inherited disorder as either an autosomal recessive or dominant inheritance, a rare pseudodominance inheritance has also been described in two families [2, 3]. Because of this, PXE is characterized by huge clinical heterogeneity in terms of the onset of the disease, the extent, and the degree of involvement of organ systems [2, 17]. However, even being a genetic disorder, the lack of familial history of PXE cannot be used to exclude the diagnosis of this condition [18]. Most of the patients develop clinical manifestations in early childhood, but cases becoming evident in late adulthood have been reported in the literature [19]. Here, our patient developed the first lesions at about the age of 16 years and none of her parents and relatives have manifested the condition until now. Although it is now believed that all cases of PXE present a familial genetic background, it is possible to speculate that PXE may also arise after a sporadic mutation, therefore culminating with the manifestation of the disease. 

In respect to the pathogenesis of the disease, it has been associated with the presence of mutation in the* ABCC6* gene [2]. *ABCC6 *gene belongs to the subfamily C of ATP-binding cassette (ABC) genes that encode a multidrug resistance-associated protein normally expressed in liver and kidneys [2]. The mutation detection varies from one study to another and has been estimated between 66 and 97% [17]. The functional association between mutated-*ABCC6* gene and PXE development is still completed unknown, especially because it was believed that the primary mutation would involve genes associated with elastic fibers synthesis, instead of a gene directly linked to a transmembrane transporter [2, 20, 21]. Otherwise, it was recently proposed that the absence of ABCC6 protein activity may lead to a reduction of inhibitors of aberrant mineralization in the peripheral tissues, which in turn are normally secreted by the liver [22].

The current classification system to support a definitive PXE diagnosis is based on the following three major criteria: (1) detectable yellowish lesions on flexural regions, (2) identification of shortened, fragmented elastic fibers as well as calcification deposits in the connective tissue on microscopic view, and (3) presence of ocular diseases, as angioid streaks, in individuals older than 20 years of old [23]. However, even considering that the majority of the cases meet all the criteria, there are reports showing PXE-affected patients who did not fulfill all the three conditions aforementioned [2, 17, 24]. For these cases, the detection of *ABCC6*-mutation gene may be useful to make a definitive diagnosis, as suggested in the literature, especially because similar alterations can be seen in other conditions, like elastosis, beta thalassemia, and Paget's disease, and a properly differential diagnosis must be made [2, 17]. Here, as our patient met all the major criteria that have been employed to confirm the PXE diagnosis, no mutational investigation of the *ABCC6* gene was performed. 

Systemic abnormalities associated with PXE are often observed in locations in which elastic fibers are the main constituent, such as the eyes and blood vessels [6, 14]. Ocular manifestations are mainly characterized by the presence of angioid streaks that may lead to central visual loss although most lesions remain stationary even over the course of the disease [2]. Several life-threatening cardiovascular conditions may affect patients with PXE including angina pectoris, arterial hypertension, and cardiac failure [2, 20]. Moreover, peripheral vessel calcification and other vascular changes may lead to gastrointestinal bleeding with the risk of death. In the present case, the patient had been previously diagnosed with PXE and had already developed chronic hypertension by the time of her initial visit to our department, and it may be associated with the disease. Angioid streaks were also detected after an ophthalmological examination, but any loss of acuity has not yet been noticed. Additionally, no other systemic abnormalities have developed during the course of the disease even more than 30 years after diagnosing. In fact, it has been reported in the literature that, due to the pattern of inheritance in PXE, it is impossible to predict the phenotype of affected patients even among members of the same family [2].

The involvement of the oral mucosa in PXE patients has been reported in the literature [7]. In an extensive review of the literature, we could find only six case reports of PXE with oral involvement, with our case representing the seventh one published until now (Table 1). Although the exact prevalence of oral lesions needs to be determined, in a French study 83% of the patients presented involvement of oral mucosa, which may be considered high when compared to another study that reported an incidence of oral lesions of only 21% [1, 9]. In the oral cavity, the lesions commonly arise inside the lower lip, but other parts, including palate and cheeks, can also be affected [25]. Because of this location, they may be misdiagnosed as Fordyce granules [7, 15]. Clinically, oral lesions appear as yellowish-white macules or papules that may have a reticular growth pattern [1]. Our patient did not develop papules on her lip but a yellowish macule, which had only appeared recently. Based on the patient's clinical signs, a biopsy was taken to confirm the condition. Light microscopy revealed a lesion composed of fragmented, granular, and calcified elastic fibers surrounded by normal connective tissue, which were histochemically confirmed by using Orcein and von Kossa stains, as described in the literature [2, 3, 16]. Although the pathogenesis of these lesions is still unknown, it has been suggested that an accentuated accumulation of proteoglycan in the PXE lesions may be involved by disturbing the assembly of the extracellular matrix and elastic fiber formation [16, 17]. The ultrastructural findings revealed electron-dense bodies deposited in the core of the elastic fibers, which can result in elastic fiber ruptures as a result of calcification or during tissue manipulation [3, 19]. All of these signs were observed in the patient's lesion and led us to confirm that her oral condition was a result of PXE. 

In conclusion, considering that PXE is a heritable multisystem disease that may be associated with a certain degree of morbidity and mortality, it is extremely important to the diagnosis of this disorder early in its course to establish a protocol for patient followup and to prevent cardiovascular, ocular, and gastrointestinal complications that may be life threatening. It is important that all PXE patients are evaluated for oral lesions. Additionally, the conditions of patients without a PXE diagnosis presenting with a yellow or white spot on the lips should not be underestimated by dental practitioners, and PXE must be considered even in the absence of skin lesions.

## Figures and Tables

**Table 1:** Case reports of PXE with oral manifestation.

Author's case	Sex	Age at presentation	Oral manifestations	Location
Matsuo et al. 2013 (present case)	Female	55 years	Yellow patches	Labial mucosa
Velazquez-Cayon et al. 2012 [8]	Female	30 years	Dental impactions	Maxilla and mandible
Morrier et al. 2008 [10]	Female	10 years	Amelogenesis imperfecta	Maxilla and mandible
Sayin et al. 2007 [11]	Female	19 years	Oligodontia, dental agenesis, and yellow patches	Maxilla, mandible, and oral mucosa
Goette and Carpenter 1981 [7]	Male	62 years	Yellow patches	Labial mucosa
Danielsen and Kobayasi 1974* [12]	?	?	?	?

*Without access to the article (cited by Goette and Carpenter 1981 [7]).
